# Cytokine Induction of VCAM-1 but Not IL13Rα2 on Glioma Cells: A Tale of Two Antibodies

**DOI:** 10.1371/journal.pone.0095123

**Published:** 2014-05-02

**Authors:** Vaidehi Mahadev, Renate Starr, Sarah L. Wright, Catalina Martinez, Michael C. Jensen, Michael E. Barish, Stephen J. Forman, Christine E. Brown

**Affiliations:** 1 Departments of Hematology and Hematopoietic Cell Transplantation, Cancer Immunotherapy & Tumor Immunology, Beckman Research Institute at the City of Hope National Medical Center, Duarte, California, United States of America; 2 Department of Neurosciences, Beckman Research Institute at the City of Hope National Medical Center, Duarte, California, United States of America; 3 Ben Towne Center for Childhood Cancer Research, Seattle Children's Research Institute, Seattle, Washington, United States of America; University of Michigan School of Medicine, United States of America

## Abstract

The interleukin-13 receptor alpha2 (IL13Rα2) is a cell surface receptor that is over-expressed by a subset of high-grade gliomas, but not expressed at significant levels by normal brain tissue. For both malignant and non-malignant cells, IL13Rα2 surface expression is reported to be induced by various cytokines such as IL-4 or IL-13 and tumor necrosis factor (TNF). Our group has developed a therapeutic platform to target IL13Rα2-positive brain tumors by engineering human cytotoxic T lymphocytes (CTLs) to express the IL13-zetakine chimeric antigen receptor. We therefore sought to investigate the potential of cytokine stimulation to induce IL13Rα2 cell surface expression, and thereby increase susceptibility to IL13Rα2-specific T cell killing. In the course of these experiments, we unexpectedly found that the commercially available putative IL13Rα2-specific monoclonal antibody B-D13 recognizes cytokine-induced VCAM-1 on glioblastoma. We provide evidence that the induced receptor is not IL13Rα2, because its expression does not consistently correlate with IL13Rα2 mRNA levels, it does not bind IL-13, and it is not recognized by IL13-zetakine CTL. Instead we demonstrate by immunoprecipitation experiments and mass spectrometry that the antigen recognized by the B-D13 antibody following cytokine stimulation is VCAM-1, and that VCAM-1, but not IL13Rα2, is induced on glioma cells by TNF alone or in combination with IL-13 or IL-4. Further evaluation of several commercial B-D13 antibodies revealed that B-D13 is bi-specific, recognizing both IL13Rα2 and VCAM-1. This binding is non-overlapping based on soluble receptor competition experiments, and mass spectrometry identifies two distinct heavy and light chain species, providing evidence that the B-D13 reagent is di-clonal. PE-conjugation of the B-D13 antibody appears to disrupt IL13Rα2 recognition, while maintaining VCAM-1 specificity. While this work calls into question previous studies that have used the B-D13 antibody to assess IL13Rα2 expression, it also suggests that TNF may have significant effects on glioma biology by up-regulating VCAM-1.

## Introduction

Malignant gliomas are highly aggressive and uniformly lethal human brain cancers for which tumor recurrence following conventional therapies remains a major challenge for successful treatment [1], [2]. Immunotherapy is emerging as a promising therapeutic approach due to its potential to specifically seek-out and attack malignant cells, particularly the infiltrated cells often responsible for disease recurrence, while sparing cells of the normal brain parenchyma. For this reason, significant efforts are dedicated towards identifying targets amenable for immunotherapy of brain tumors.

One attractive immunotherapy target is IL13Rα2, a 42-kDa monomeric high affinity IL-13 receptor distinct from the more ubiquitously expressed IL-13Rα1/IL-4Rα receptor complex [3]. IL13Rα2 is expressed by a high percentage of gliomas, but not at significant levels on normal brain tissue [4]–[7], and in IL13Rα2-expressing tumors has been identified on both stem-like malignant cells and their more differentiated counterparts [8]. Targeting IL13Rα2 is currently the focus of ongoing clinical development for the treatment of brain tumors [8]–[12]. In one such effort, our group has constructed an IL13 (E13Y)-zetakine CAR for targeting IL13Rα2. Expanded *ex vivo*, IL13(E13Y)-zetakine^+^ CTL retain MHC-independent IL13Rα2-specific anti-glioma cytolytic activity, maintain CAR-regulated Tc1 cytokine secretion and proliferation, and mediate regression of established human glioblastoma xenografts *in vivo* [12]. These pre-clinical studies have culminated in a FDA-authorized feasibility/safety clinical trial of intracranial adoptive therapy with autologous IL13-zetakine^+^ CD8^+^ CTL clones targeting recurrent/progressive malignant glioma.

Because various combinations of cytokines (i.e., TNF, INFγ, IL-4 and IL-13, and combinations thereof) have been reported to induce IL13Rα2 on a variety of cell types [13]–[15], we reasoned that using similar protocols to increase surface expression of IL13Rα2 on glioma cells would enhance therapeutic efficacy of multiple IL13Rα2-targeting treatment modalities including IL13(E13Y)-zetakine^+^ CTLs.

However, in the course of these studies we obtained divergent results with two IL13Rα2-directed antibodies: a goat polyclonal antibody from R&D Systems (cat# AF146) and a PE-conjugated mouse monoclonal antibody clone B-D13 from Cell Sciences. In reconciling these observations, we determined that the putative IL13Rα2-specific antibody B-D13 recognizes VCAM-1, and that cytokine induction is not a viable approach to increase cell surface expression of IL13Rα2 for therapeutic targeting of gliomas. Instead, we find that cytokine stimulation induces VCAM-1 expression by glioma cells, an observation of potential significance for understanding cytokine influences on glioma progression and dissemination.

## Methods

### Cell lines and culture conditions

The human monocytes line THP-1, glioblastoma line T98, medullablastoma line D283, and SV40 T antigen transformed human embryonic kidney line 293T were obtained from ATCC. The glioma line U251 originated from ATCC, and was a gift from Dr. Waldemar Debinsky (Wake Forest School of Medicine), and after being verified as tumorigenic designated U251T. D283 cells were engineered to express full length, human IL13Rα2 using lentiviral transduction. 293T cells were transiently tranfected using lipofectamine 2000 reagent (Invitrogen) to express either full length VCAM-1 (OriGene) or IL13Rα2 (Geneart). Primary glioma lines were derived from patients undergoing tumor resections at City of Hope. In some cases tumor explants were expanded by heterotopic subcutaneous (s.c.) passaging in mice prior to growth and characterization in culture; in such cases the s.c. passage number is reported after the PBT number (e.g., PBT003-4). Primary brain tumor lines were cultured in neural stem cell medium [DMEM:F12 (Irvine Scientific), 1∶50 B27 (Invitrogen), 5 ug/mL heparin (Abraxis Pharmaceutical Products), and 2 mmol/L L-glutamine (Irvine Scientific)]. U251T and T98 cells were cultured in DMEM (high glucose; Gibco) supplemented with 10% heat inactivated fetal bovine serum (Omega Scientific), and 1% L-glutamine (Gibco).

For induction of the B-D13 antigen, cells were induced overnight (16–20 hours) with either 20 ng/mL of TNF-α (Peprotech) alone or in combination with 20 ng/mL IL-4 or IL-13 (Peprotech). To block transcription, cells were incubated with varying concentrations of Actinomycin D for 1.5 hours followed by incubation with 20 ng/mL of TNF/IL-4 for 5 hours. To block translation, cells were incubated with varying concentrations of Cyclohexamide for 3 hours followed by incubation with 20 ng/mL of TNF for 5 hours.

### Flow cytometry and Western analysis

Cell surface expression was evaluated by staining with phycoerythrin (PE)-conjugated anti-VCAM-1 (eBioscience, 12-1069-41), goat polyclonal anti-IL13Rα2 AF146 (R&D Systems) followed by detection with PE-conjugated donkey anti-goat (Novus Biologicals), PE-conjugated mouse monoclonal anti-IL13Rα2 B-D13 (Cell Sciences; lots 1107403, 1107404 and 1107405), or unconjugated B-D13 antibodies (Cell Sciences, Abcam and Santa Cruz) followed by detection with PE-conjugated goat anti-mouse (Jackson ImmunoResearch). In some cases B-D13 reagent was first pre-incubated for thirty minutes with soluble recombinant human IL13Rα2-Fc chimera or VCAM-1/CD106-Fc chimera reagents (R&D Systems). Percentages of positively staining cells were calculated using the subtraction method with FCS Express version 3 software (De Novo Software, Los Angeles, CA). Relative fluorescence intensity was calculated by dividing the MFI experimental by the MFI of the control. IL-13 biotin binding assays were done by biotinylating IL-13 (Thermoscientific) at a 2.5 biotin to recombinant IL-13 ratio using the EZ-Link Sulfo-NHS-Biotin kit (Thermoscientific) as per the manufacturer's instructions. The biotinylated IL-13 was then incubated with various glioma lines, followed by staining with streptavidin-PE.

For immunoprecipitation assays, PBT008 and THP-1 cells were stimulated with cytokines as indicated and then incubated with or without unconjugated B-D13 antibody (Cell Sciences; 5 µg antibody per 8×10^6^ cells), washed 3 time in PBS (Irvine Scientific) to remove excess antibody. Cells were then lysed in RIPA assay buffer (PBS, 0.5% sodium deoxycholate, 0.1% SDS, 0.5% Igepal) and antibody/protein complexes were precipitated with Protein G beads. The bound antigen was gently eluted from the beads in 0.3 M glycine pH 2.8, in a series of elutions (E1, E2, E3).

For Western analysis, protein extracts were diluted in 1 mM BME-containing (Bio-Rad) Laemmli loading buffer (Bio-Rad) and separated on 4%–12% gradient SDS-PAGE gels (Bio-Rad) nitrocellulose membrane and probed with the indicated antibodies.

### qPCR

To quantify mRNA levels of various genes, RNA samples were isolated from cells using the RNeasy Kit (Qiagen), and cDNA were prepared using the Superscript First Strand Synthesis System for reverse transcription PCR (RT-PCR) (Invitrogen). Real time PCR analysis was done using a custom plate from RT^2^ Custom Profiler PCRArray CAPA9696-24 (SABiosciences) and analyzed using the Web-based PCR Array Data Analysis. Fold changes in mRNA levels were calculated after normalization to housekeeping genes [β-Actin (ACTB), glyceraldehyde-3-phosphate dehydrogenase (GAPDH), ubiquitin C (UBC), 60 S acidic ribosomal protein P0 (RPLP0)].

### Chromium Release Assay

The cytolytic activity of T-cell effectors was determined by 4-hour chromium release assay [16]. Briefly, 5×10^3^ ^51^Cr- labeled target cells (Na_2_^51^CrO_4_; (5 mCi/mL); Amersham Parmacia, Piscataway, NJ) seeded into triplicate wells containing the effector cells at various E:T cell ratios in 200 µL of DMEM supplemented with 10% FCS were seeded into V-bottom 96-well micro-plated and incubated for 4 hours at 5% CO_2_, 37°C. Plates were centrifuged, and 100 µl of supernatant was removed from each well to assess chromium release using a γ-counter (Packard Cobra II, Downer's Grove, IL). The percentage of specific lysis (%) was calculated as follows: 100× (experimental release – spontaneous release)/(maximum release – spontaneous release). Maximal release represents the radioactivity released after total lysis of target cells with 1% SDS. Spontaneous release represents the radioactivity released by target cells present in the medium without effector cells.

### Mass Spectrometry

#### Protein identification

*Protein digestion*: Proteins were denatured in 1∶1 v/v trifluoroethanol (TFE), reduced, alkylated and digested with ChymoTrypsin (Promega) according to published protocols [17].

*LCMS/MS analysis*: An Agilent 6520 QTOF equipped with an Agilent chip-cube nano-electrospray interface and an Agilent 1200 nano-LC system was used: Samples were trapped on an Agilent High capacity Chip (part #: G4240-62010) at 4 uL/min 99% buffer A (0.1% formic acid in water) and 1% buffer B (0.1% formic acid in acetonitrile), then eluted at 0.3 uL/min from 1% to 85% buffer B. The QTOF was operated in Auto MS/MS mode with the standard parameters for chip cube interface. MS spectra were acquired from 300 to 2500 m/z and MS/MS was on the six most intense ions in each MS scan, in the range 300–3000 m/z.

*Data analysis*: Data was searched using X-Tandem! (version: TORNADO 2009.04.01.1). The following parameters were used: Fragment Tolerance: 50 PPM (Monoisotopic), Parent Tolerance: 25 PPM (Monoisotopic), Fixed Modifications: +57 on C (Carbamidomethyl), Variable Modifications: +1 on NQ (Deamidation), +16 on MW (Oxidation), +32 on M (Sulphone), +42 on n (Acetyl), Database: the Human Ensembl database and crap.fasta.pro database (unknown version, 76704 entries), Digestion Enzyme: Chymotrypsin, Max Missed Cleavages: 3

#### Antibody characterization

Antibodies were reduced by mixing the antibody solution 1∶1 with 100 mM dithiothreitol (Sigma) in 1 M ammonium bicarbonate and incubating for 1 hour at 57°C. Reduced antibodies were analyzed by LC/MS using the same mass spectrometer, HPLC, and source, but with an Agilent intact protein chip (part# G4240-63001 SPQ105). Antibodies were loaded onto the trapping column at 6 µl/min in 1% Buffer B, then eluted through the analytical column with a linear gradient from 1% to 99% Buffer B. Multiply-charged protein spectra were deconvoluted using Agilent Bioconfirm software to give the uncharged protein masses.

## Results

### Differential recognition of cytokine-induced epitopes by two IL13Rα2-directed antibodies

**Figure 1:**
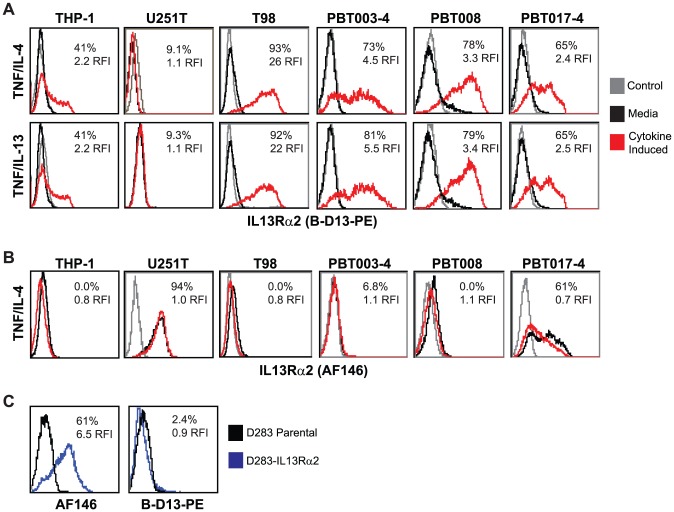
Differential recognition of constitutively-expressed versus cytokine-induced IL13Rα2 by commercially available anti-IL13Rα2 antibodies. Flow cytometric analysis of monocytic line THP-1 and various glioma lines with (A) B-D13-PE (Cell Sciences) or (B) AF146 (R&D Systems) reagents for media alone (black histogram) and cytokine (TNF/IL-4 or TNF/IL-13 overnight; red histogram) conditions. Isotype (iso-PE) and mouse anti-goat-FITC controls are shown as grey histograms. Percent positive and relative fluorescent index (RFI) of MFI cytokine/MFI media is reported for each histogram. (C) Flow cytometric detection of IL13Rα2 for D283 cells engineered to express IL13Rα2 (D283-IL13Rα2; blue histogram) and D283 parental (black histogram) stained with AF146 or B-D13-PE antibodies. All data are representative of more than three experiments each.

We investigated the possibility of up-regulating cell surface IL13Rα2 levels on glioma utilizing cytokine stimulation regimens previously reported to induce IL13Rα2 on other cell types [13]–[15], including the monocytic cell line THP-1 [13]. Indeed, using the commercially available PE-conjugated B-D13 mouse monoclonal antibody (B-D13-PE; Cell Sciences) reported to target IL13Rα2, we find that cell surface antigen expression on negative or low expressing glioma cell lines (T98, PBT003-4, PBT008 and PBT017-4) is up-regulated following incubation with either TNF and IL-4, or TNF and IL-13 (Figure 1A). Induction of the B-D13-PE target antigen was observed on all the glioma cell lines except U251T, to levels equivalent or greater than observed with the THP-1 line used as a positive control [13]. Further, B-D13-PE target antigen was up-regulated on glioma cell lines under a variety of cytokine conditions including TNF alone (Figure S1).

We have previously demonstrated that the goat polyclonal anti-IL13Rα2 antibody AF146 (R&D Systems) specifically recognizes human IL13Rα2 as shown by FACs, Western and qPCR [8]. However, inconsistencies were noted using B-D13-PE antibody that led us to consider the possibility of multiple IL13Rα2 isoforms or epitopes, or, alternatively, the induction of a different molecule recognized by B-D13-PE. First, the U251T glioma cell line does not express the B-D13-PE recognized antigen (Figure 1A), even though this glioma cell line is known to express high levels of IL13Rα2 [8]. Secondly, AF146 antibody did not recognize the cytokine induced putative IL13Rα2 antigen detected by B-D13-PE antibody on any of the cell lines evaluated (T98, PBT003-4, PBT008, PBT017-4 and THP-1) (compare Figure 1A to 1B). Interestingly, while most of the cell lines displayed mutually exclusive expression of the B-D13-PE and AF146 antigens, PBT017-4 expressed IL13Rα2 as detected by AF146 [8], and, following cytokine stimulation, also showed independent induction of the B-D13-PE antigen. Finally, the AF146 antibody recognizes cells engineered to express IL13Rα2 (D283- IL13Rα2), whereas B-D13-PE does not (Figure 1C). These findings clearly demonstrate the differential recognition of constitutive IL13Rα2 (expressed by U251T and D283-IL13Rα2) and the cytokine-induced antigen (up-regulated on THP-1, PBT003-4, PBT008 and PBT017) by B-D13-PE as compared to the IL13Rα2-validated antibody AF146.

### The cytokine induced antigen recognized by the B-D13 antibody is not IL13Rα2

Our observations led us to evaluate this discrepancy in IL13Rα2 recognition in more detail. Several lines of evidence, including evaluation of IL13Rα2 mRNA levels and IL-13 binding studies, suggest that the cytokine-induced antigen recognized by the B-D13-PE antibody is a molecule distinct from IL13Rα2.

#### Cytokine-induced B-D13 antigen expression does not correlate with changes in IL13Rα2 mRNA levels

**Figure 2:**
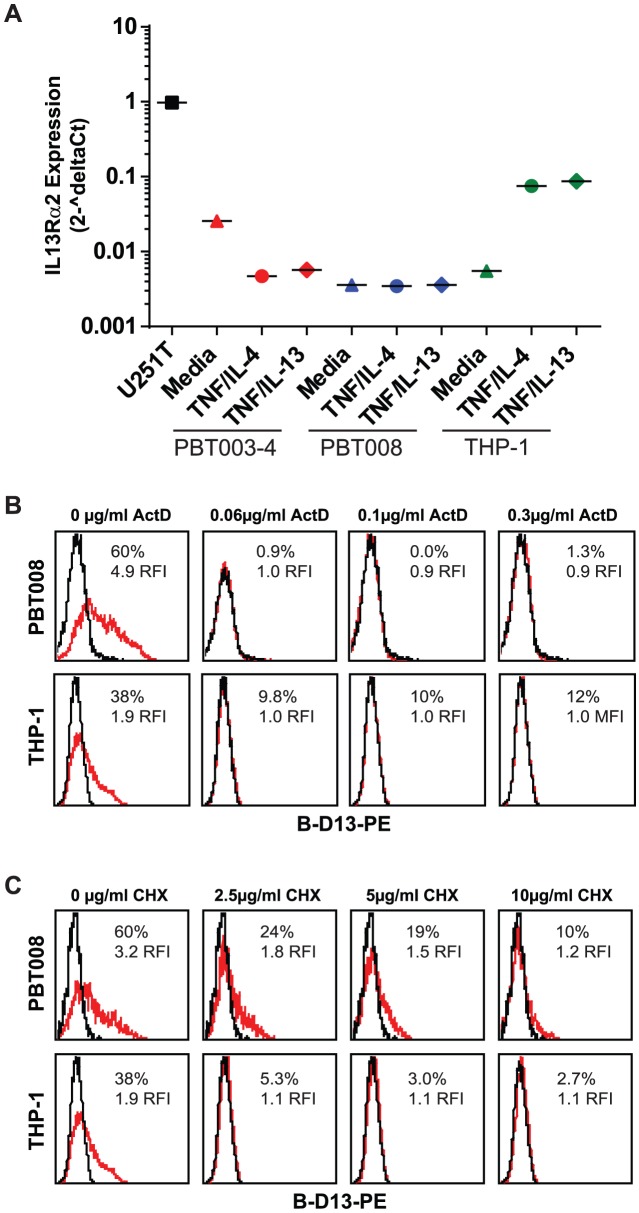
Cytokine induction of B-D13 antigen is dependent on mRNA transcription and translation. (A) IL13Rα2 mRNA levels quantified by qPCR for U251T, PBT003-4, PBT008, and THP-1 cells after overnight culture in media, TNF/IL-4, or TNF/IL-13. mRNA levels were normalized to housekeeping genes (ACTB, UBC, GAPDH and RPLP0). (B, C) B-D13-PE immunoreactivity of PBT008 and THP-1 cells treated with increasing concentrations of (B) transcription blocker Actinomycin D (ActD) (0, 0.06, 0.1, 0.3 µg/ml) or (C) translation blocker Cycloheximide (CHX) (0, 2.5, 5, 10 µg/ml), then either cultured in media alone (black histograms) or stimulated with TNF/IL-4 (red histograms) for 5 hours. All data are representative of 2 separate experiments.

We reasoned that if the cytokine-induced B-D13-PE target antigen was an isoform of IL13Rα2, then IL13Rα2 mRNA levels would also increase upon cytokine stimulation when analyzed by qPCR. We focused on THP-1, PBT003 and PBT008 because these cell lines exhibited high B-D13-PE target antigen up-regulation following cytokine stimulation. Under basal conditions, all three cell lines expressed significantly lower levels of IL13Rα2 mRNA as compared to IL13Rα2-expressing U251T cells: 0.5% for THP-1, 0.3% for PBT003 and 2% for PBT008 cells (Figure 2A). Following cytokine stimulation, IL13Rα2 mRNA levels went down for PBT003 and did not increase in PBT008 cells, despite the significant increase in cell surface B-D13-PE target antigen expression detected by flow cytometry (PBT003: MFI increased 19-fold (RFI); PBT008: MFI increased 28-fold (RFI)) (Figure 1A). We did detect an increase in IL13Rα2 mRNA levels for the THP-1 monocyte line (13–15 fold increase; Figure 2A) consistent with previous reports [13]. However, IL13Rα2 mRNA levels for THP-1 cells after cytokine stimulation were more than 13–15 fold lower than for steady state U251T cells (Figure 2A), and induced IL13Rα2 cell surface expression was below the limit of antibody AF146 detection (Figure 1B). Thus, by qPCR, induction of B-D13-PE target antigen does not consistently correlate with IL13Rα2 mRNA levels.

To rule out the possibility that discordance of cytokine induction of the B-D13-PE target antigen with mRNA levels was due to pre-existing mRNA or intracellular stores that could be mobilized to the cell surface following cytokine stimulation independently of transcription or translation, we evaluated the sensitivity to inhibitors of mRNA or protein synthesis [14]. To test a potential requirement for mRNA synthesis, we treated THP-1 and PBT008 cells for 1.5 hours with increasing concentrations of the transcription blocker actinomycin D, followed by 5 hours cytokine stimulation with TNF plus IL-4. Induction of the B-D13-PE target antigen was strongly inhibited by 0.06 ug/ml actinomycin D treatment (Figure 2B). Similar inhibition was observed by blocking protein synthesis with cycloheximide (completely blocked at 10 µg/ml; Figure 2C). These observations suggest that cytokine stimulation did not mobilize pre-existing B-D13-PE target antigen, and that new transcription was required for the observed B-D13-PE antigen induction.

#### The cytokine-induced B-D13 antigen does not bind IL-13 and is not recognized by IL13Rα2-redirected CTLs

**Figure 3:**
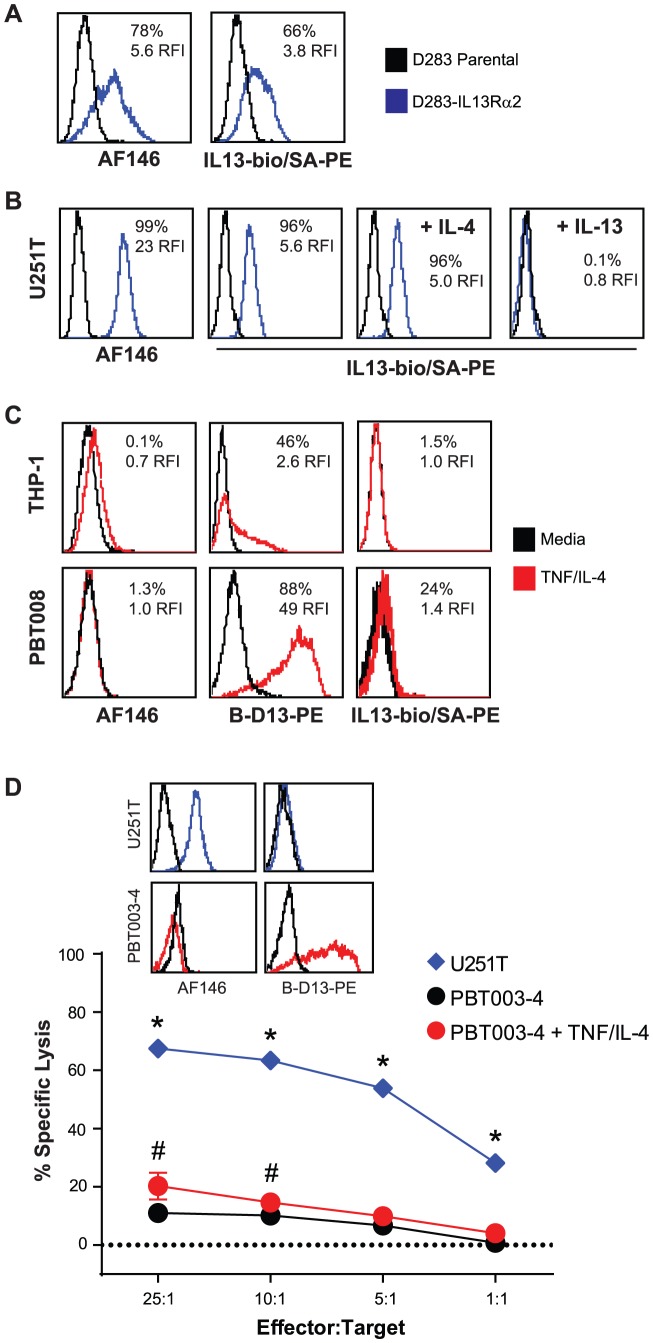
The cytokine-induced cell surface antigen recognized by B-D13-PE does not bind IL-13. (A) D283 cells engineered to express IL13Rα2 (D283- IL13Rα2) (blue histograms) and D283 parental (black histograms) cells were evaluated by flow cytometry for expression of constitutive IL13Rα2 using the IL13Rα2-specific antibody AF146, and biotinylated recombinant human IL-13 (IL13-bio) followed by PE-conjugated strepavidin (SA-PE). Data are representative of 2 separate experiments. (B) U251T (grown in the absence of cytokines) were evaluated by flow cytometry for constitutive IL13Rα2 expression using AF146, and for binding to IL13-bio/SA-PE in the presence and absence of 10-fold molar excess of recombinant human IL-4 or IL-13. Black histograms represent staining with istoype control antibody or SA-PE alone. Data are representative of 2 separate experiments. (C) THP-1 and PBT008 grown in media alone (black histograms) or induced overnight with TNF and IL-4 (red histograms) were analyzed by flow cytometry for expression of constitutive IL13Rα2 (AF146), for expression of the induced antigen (B-D13-PE), and for binding to IL13-bio/SA-PE. Data are representative of 3 separate experiments. (D) IL13-zetakine^+^ CD8^+^ CTL recognize and kill U251T glioma targets expressing constitutive IL13Rα2 (AF146-positive), but not cytokine-induced PBT003 cells (B-D13-positive). Percentage specific lysis (mean ± S.D.) of triplicate wells is depicted. *, p≤0.0002 using an unpaired Student's t-test to compare U251T vs. PBT003-4 targets. #, p>0.05 using an unpaired Student's t-test to compare PBT003-4 targets with and without overnight cytokine stimulation. Data are representative of at least 2 separate experiments.

We tested whether the cytokine-induced B-D13-PE target antigen binds IL-13 using a biotinylated IL-13 binding assay. Biotinylated human recombinant IL-13 binds to D283 cells engineered to express IL13Rα2 (D283-IL13Rα2), but not the IL13Rα2-negative parental control (Figure 3A), and AF146 immunoreactivity follows this pattern. IL13Rα1 heterodimerizes with IL4Rα and binds both IL-13 and IL-4, whereas IL13Rα2 selectively binds only IL-13 but not IL-4 [14]. To confirm authentic IL13Rα2 expression, we demonstrated that biotinylated IL-13 bound to IL13Rα2-expressing U251T cells, and is competed off by pre-incubation with 10-fold molar excess of unconjugated IL-13, but not by preincubation with the same molar excess of IL-4 (Figure 3B). In contrast, cytokine-stimulated THP-1 and PBT008 cells do not bind biotinylated IL-13 despite high cell surface induction of the B-D13 target antigen (Figure 3C). These experiments indicate that cells expressing IL13Rα2 which are reactive with AF146 antibody bind biotinylated IL-13, while cytokine-induced cells expressing the B-D13-PE target antigen fail to show biotinylated IL-13 binding.

In an independent approach to evaluate IL-13 binding, we used a highly sensitive cytotoxicity assay to determine whether IL13Rα2-specific redirected CTLs recognize the B-D13-PE target antigen. We have previously shown that IL-13-zetakine T cell lines specifically lyse IL13Rα2-positive target cells in an MHC-independent manner [8], [12]. As expected, the IL13Rα2-positive U251T line is efficiently killed by IL13-zetakine+ T cells (Figure 3D). In contrast, IL-13-zetakine+ T cells did not recognize and kill PBT003 cell lines above background following cytokine induction of the B-D13-PE target antigen.

Taken together, the discordance between cytokine induction of B-D13-PE target antigen and IL13Rα2 mRNA levels, along with the demonstration that the cytokine-induced B-D13-PE target antigen does not bind IL-13 and is insensitive to IL13Rα2-directed killing, suggest that the inducible molecule is not IL13Rα2.

### B-D13 antibody recognizes VCAM-1

**Figure 4:**
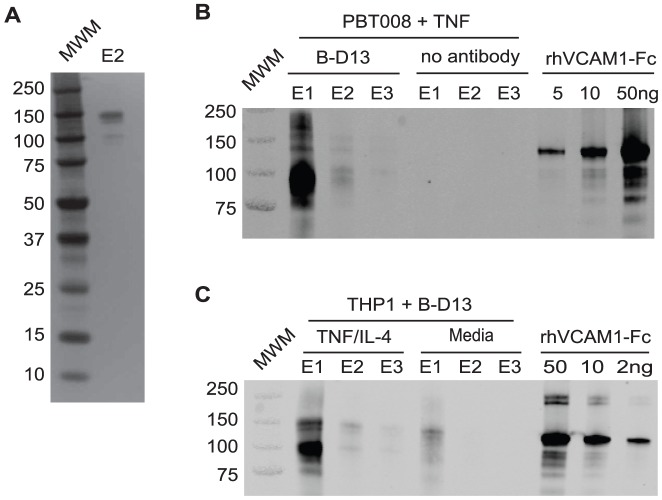
The B-D13 antibody recognizes VCAM-1, not IL13Rα2. (A) Silver stain gel of the second elution (E2) from the B-D13 immunoprecipitation of PBT008 stimulated overnight with TNF. (B) Western blot detecting B-D13 pull-down of VCAM-1 in immunoprecipitation eluates (E1, E2, E3) for PBT008 cells stimulated overnight with TNF. VCAM-1 was not immunoprecipitated in the absence of B-D13 antibody (no antibody: E1, E2, E3). Titrated soluble recombinant human VCAM-1-Fc shows specificity of the VCAM-1 antibody. (C) Western blot detecting B-D13 pull-down of VCAM-1 in immunoprecipitation eluates (E1, E2, E3) for THP-1 cells in both media and cytokine overnight-stimulated conditions. All data are representative of 2 separate experiments.

To identify the cytokine-induced B-D13 target antigen we performed protein immunoprecipitation followed by tandem mass spectrometry. We focused on PBT008 glioma cells because this cell line displayed strong cell surface induction of the B-D13-PE target antigen. Following overnight cytokine stimulation with TNF, intact cells were incubated with B-D13 antibody (unconjugated antibody; Cell Sciences), lysed and antigen-antibody complexes immunoprecipitated using Protein G beads. Immunoprecipitated proteins were eluted in low-pH buffer in three fractions (E1, E2 and E3), and a silver stained gel of E2 detected three distinct polypeptides between 100 to 150 kDa (Figure 4A). To identify the immunoprecipitated polypeptides, protein eluates were subjected to LC/MS/MS and in two independent experiments mass spectrometry identified the B-D13 immunoprecipitated protein as VCAM-1 (IP #1: 3 peptides; IP #2: 13 peptides). The only other proteins detected in the eluates were either present in the control Protein G IP performed without B-D13 antibody or expected contaminants such as keratin and chymotrypsin (Table 1).

**Table 1:** Peptides identified by tandem mass spectrometry.

Protein	Molecular Weight (kDa)	Protein Identification Probability	Number of Unique Peptides	Number of Unique Spectra	Total Number of Spectra	Percent Sequence Coverage
Vascular cell adhesion molecule 1	81.3	100.00%	13	13	20	25.40%
Chymotrypsinogen A	25.7	100.00%	13	18	37	45.70%
Immunoglobulin kappa variable 4-1	13.4	100.00%	1	2	3	9.92%
Heat shock 70 kDa protein 9	73.6	100.00%	2	2	2	8.84%

To confirm that the protein product immunoprecipitated by B-D13 antibody is VCAM-1, Western analysis was performed on the immunoprecipitated eluates. For cytokine-induced PBT008 cells, the B-D13 antibody pulled-down polypeptides ranging from 100 to 150 kD protein which were immunoreactive for VCAM-1 (Figure 4B). Immunoprecipitation of VCAM-1 was specific, as it was not immunoprecipitated from cytokine induced (TNF) PBT008 cells in the absence of B-D13 antibody (Figure 4B; see no antibody E1-E3). For THP-1 cells, the B-D13 antibody also immunoprecipitated the VCAM-1 immunoreactive antigen following cytokine induction (TNF/IL-4), and VCAM-1 was immunoprecipitated at significantly lower levels in non-cytokine exposed cells (media) (Figure 4C). Although THP-1 cells did show an increase in IL13Rα2 mRNA levels following cytokine induction (13–15 fold, Figure 2A), the B-D13-immunoprecipitated protein was not recognized by the IL13Rα2-specific antibody AF146 (not shown).

**Figure 5:**
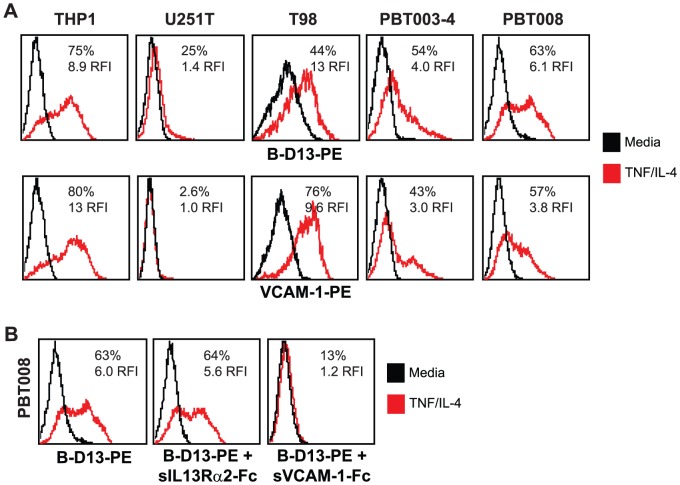
Antibody immunoreactivity, and soluble ligand competition confirms B-D13-PE specificity for VCAM-1. (A) Flow cytometric analysis of monocytic line THP-1 and various glioma lines with B-D13-PE (top), or PE-conjugated anti-VCAM-1 (VCAM-1-PE, bottom), after overnight culture in media alone (black histogram) versus cytokine (TNF/IL-4; red histogram) conditions. Data are representative of at least 2 separate experiments. (B) PBT008 cells cultured overnight in media alone (black histogram) versus cytokine (TNF/IL-4; red histogram) conditions were stained with B-D13-PE antibody that was pre-incubated with soluble recombinant human IL13Rα-Fc or VCAM-1-Fc. As a control, cells were stained with B-D13-PE alone. Data are representative of 2 separate experiments.

Further, authentic VCAM-1 immunoreactivity correlated with B-D13-PE staining on all lines exhibiting cytokine-induced antigen expression (Figure 5A, see THP-1, T98, PBT003-4, and PBT008), while cytokine-stimulated U251T cells showed neither induction of VCAM-1 nor B-D13-PE immunoreactivity. In contrast, and consistent with our previous findings (Figure 1), no induction of IL13Rα2, as detected by the AF146 antibody, was seen on any of the lines responding to TNF/IL-4, and U251T cells expressed constitutively high levels of IL13Rα2.

The VCAM-1 specificity of B-D13-PE was confirmed by ligand competition, in which we compared the potential of either soluble recombinant human VCAM-1-Fc (sVCAM-1-Fc) or IL13Rα2-Fc (sIL13Rα2-Fc) to compete for B-D13-PE immunoreactivity on TNF/IL-4-stimulated PBT008 cells (Figure 5B). Pre-incubation of sVCAM-1-Fc, but not sIL13Rα2-Fc, inhibited B-D13-PE antibody binding on cytokine induced PBT008 cells, indicating that soluble VCAM-1 blocks the binding site for the epitope recognized by antibody B-D13-PE.

Thus by four independent measures — mass spectrometry, Western analysis, direct comparison of antibody immunoreactivity, and soluble ligand competition — the protein induced by cytokine (TNF, TNF/IL-4 or TNF/IL-13) and recognized by antibody B-D13-PE was identified as VCAM-1.

### Characterization of additional commercially available B-D13 antibodies

**Figure 6:**
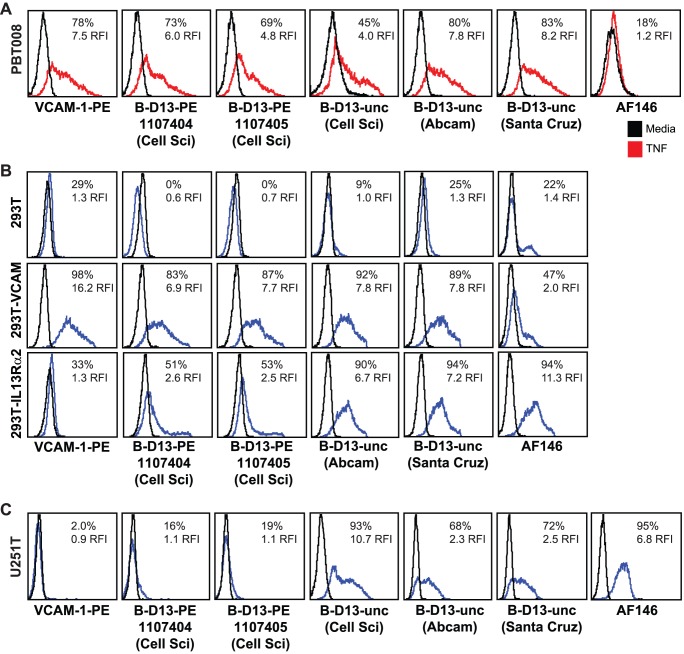
Evaluation of additional commercially available B-D13 antibodies. (A) PBT008 cells that had been cultured overnight in media alone (black histogram) versus cytokine (TNF; red histogram) conditions, (B) Parental 293T cells or 293T cells engineered to express either VCAM-1 or IL13Rα2, and (C) U251T cells were stained with VCAM-1-PE, AF146 or various B-D13 reagents – two lots of PE-conjugated B-D13 antibody (B-D13-PE; Cell Sciences) and two to three unconjugated B-D13 antibodies (B-D13-unc) purchased from either Cell Sciences (Cell Sci), Abcam or Santa Cruz as indicated. (B, C) Black histograms represent staining with istoype control antibody or SA-PE alone.

To provide further insight into the specificity discrepancies revealed by our studies for the B-D13 antibody, we compared the behaviors of two lots of PE-conjugated B-D13 (B-D13-PE; Cell Sciences lots 1107404 and 1107405), as well as unconjugated B-D13 antibodies (B-D13-unc) from Cell Sciences, Santa Cruz and Abcam. We confirmed by flow cytometric analysis that each commercially available B-D13 reagent — two B-D13-PE lots and three B-D13-unc antibodies — stained cytokine-stimulated PBT008 cells in a manner similar to that of the VCAM-1 specific antibody (Figure 6A). These B-D13 reagents also recognized 293T cells engineered to express VCAM-1 by transient transfection, but not parental 293T cells (Figure 6B, top and middle panels). Thus recognition of VCAM-1 by the B-D13 antibodies is independent of antibody lot or commercial source.

Interestingly, however, the unconjugated B-D13 antibodies (B-D13-unc) also recognized 293T cells transiently transfected to express IL13Rα2, as well as constitutively IL13Rα2-expressing U251T cells (Figure 6B lower panels, and 6C). This is in contrast to the two lots of PE-conjugated B-D13 from Cell Sciences that did not strongly recognize IL13Rα2 on either U251T or IL13Rα2-engineered 293T cells. These findings suggest that PE-conjugation impairs IL13Rα2 recognition, and that unconjugated B-D13 antibody, independent of commercial source, recognizes both IL13Rα2 and VCAM-1.

**Figure 7:**
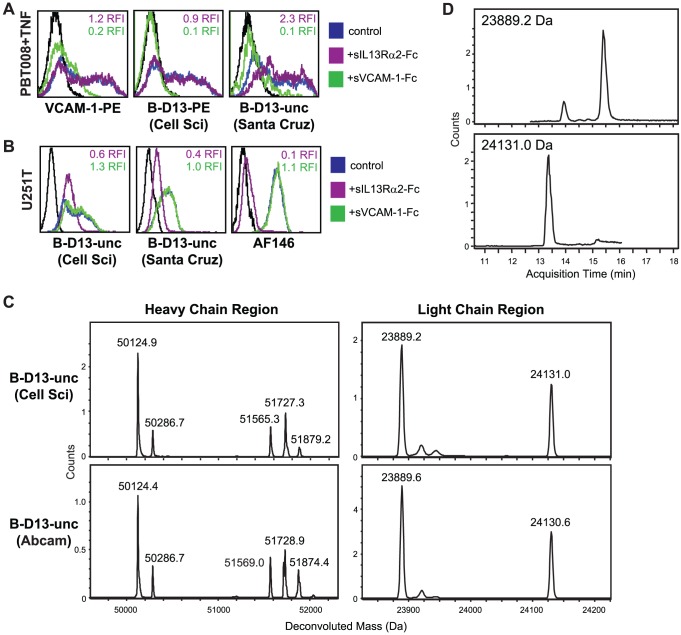
B-D13 reagent appears to contain two distinct monoclonal antibodies. Soluble receptor competition study evaluating the specificity of IL13Rα2 and VCAM-1 recognition by the B-D13-unc antibodies (Cell Sciences and Santa Cruz) using (A) PBT008 cells cultured overnight in media alone (black histogram) versus cytokine (TNF; blue histogram); or (B) IL13Rα2-expressing U251T cells (blue histogram). Cells were stained with the indicated antibody that was pre-incubated with soluble recombinant human IL13Rα-Fc (purple histograms) or VCAM-1-Fc (green histograms). Relative fluorescence index (RFI) compared to staining without the soluble competitors (i.e., the control/blue histograms) are indicated in each histogram. (C) Unconjugated B-D13 antibodies from Cell Sciences (top) and Abcam (bottom) were reduced and analyzed by LC/MS. Shown is the spectra of the deconvoluted protein masses depicting two distinct mass species for both the heavy and light chains. (D) Extracted ion chromatograms (EIC) for the two light chain species, of the Cell Sciences B-D13-unc reagent from (C).

We next set out to understand the bi-specificity of the B-D13-unc antibodies, and the results of several experiments suggest the presence of two distinct monoclonal antibodies in the B-D13 preparation, which recognize either IL13Rα2 or VCAM-1. First, in competition experiments, only soluble VCAM-1-Fc, but not soluble IL13Rα2-Fc, could compete for recognition of VCAM-1 on cytokine-induced PBT008 cells (Figure 7A). Reciprocally, only soluble IL13Rα2-Fc, but not soluble VCAM1-Fc, could compete for recognition of IL13Rα2 on U251T cells (Figure 7B). These results demonstrate that the IL13Rα2 and VCAM-1 antigen binding sites of the B-D13-unc antibody are non-overlapping. Second, mass spectrometry analysis identified two distinct heavy and light chains in the B-D13-unc antibody preparations, that were identical between those purchased from either Cell Sciences or Abcam (Figure 7C) (B-D13-unc from Santa Cruz was not evaluated due to the presence of 0.1% gelatin in the preparation that would interfere with this analysis). The deconvoluted mass of the two heavy chains differed by more than 1,500 Da (e.g., 50124.9 vs. 51565.3 for the Cell Sciences B-D13-unc reagent), and the multiple mass species for each (i.e., the clusters of peaks) reflect differences in glycosylation states (Figure 7C). The deconvoluted mass of the two light chains differed by more than 240 Da (e.g., 23889.2 vs. 24131.0 for the Cell Sciences B-D13-unc reagent; Figure 7C), and further were chromatographically separated by HPLC with a retention time difference of approximately 2 minutes, which is indicative of differential amino acid composition and/or hydrophobicity (Figure 7D). Taken together, these data are most consistent with the B-D13 antibody reagent being di-clonal due to the presence of two distinct monoclonal antibodies in the preparation.

## Discussion

These experiments were prompted by efforts to use cytokine-stimulation paradigms to increase IL13Rα2 expression on glioma cells and thereby increase the efficacy of IL13Rα2 targeted therapies for brain tumors [8]–[12]. Based on the many studies which reported induction of IL13Rα2 on a variety of cell types following cytokine stimulation [13]–[15], we envisioned that this strategy for IL13Rα2 induction may be conserved for glioblastoma as well. Indeed, following cytokine stimulation, we did observe induction of a cell surface antigen on both primary glioblastoma cell lines and the monocyctic cell line THP-1, which was strongly detected by the commercially available putative IL13Rα2-targeted monoclonal antibody B-D13-PE (Cell Sciences). However, during the course of these studies, we encountered a series of discrepancies in the behavior of the B-D13-PE antibody that led us to question its binding specificity, and whether the induced antigen following cytokine stimulation was really IL13Rα2. In particular, following cytokine stimulation, B-D13-PE immunoreactivity did not correlate with either the immunoreactivity of the highly-characterized IL13Rα2-specific goat polyclonal antibody AF146 (R&D Systems) [18] or IL13Rα2 mRNA levels. Further, the cytokine induced B-D13-PE antigen did not bind IL-13 or elicit lysis by IL13Rα2-redirected CAR T cells, and B-D13-PE binding on cytokine stimulated cells could not be blocked by soluble IL13Rα2-Fc. Therefore, our data indicate that neither TNF/IL-4, TNF/IL-13, nor TNF alone induce cell surface IL13Rα2 up-regulation on glioma cells (or at significant levels on monocytes), and therefore that these cytokine treatments are not a viable strategy for expanding the targetability of IL13Rα2 by immunotherapy.

Instead, our data definitively demonstrate that the cytokine induced antigen recognized by B-D13-PE is VCAM-1, as demonstrated by B-D13 immunoprecipitation/mass spectrometry, as well as soluble receptor competition studies. Many studies have reported induction of IL13Rα2 on a variety of cell types following cytokine stimulation [13]–[15], and induction of IL13Rα2 has been reported to be involved in TGF-β_1_ production [13], [15], [19]. However, all of these studies used the B-D13 antibody to evaluate protein induction, and thus may have inadvertently mis-identified the induction of IL13Rα2 protein following cytokine stimulation. It should be noted, however, that qPCR and knockdown studies do support IL13Rα2 induction in some cell types [13]. In fact, consistent with previous reports [13], we find that THP-1 cells show induction of IL13Rα2 mRNA 13 to 15-fold after overnight treatment with TNF and IL-13 or IL-4, although the level of IL13Rα2 expression was more than 13.6-fold lower than that expressed by the U251T glioma cell line and not at sufficient levels to be detected by flow cytometry using the IL13Rα2-specfic AF146 antibody. We do not, however, find any evidence for induction of IL13Rα2 through TNF/IL-4 or TNF/IL-13 cytokine regimens on glioma cell lines, either established (T98 or U251T) and/or low-passage primary lines (PBT003-4, PBT008, PBT017-4).

The B-D13 antibody has also been used to evaluate constitutive IL13Rα2 expression on a variety of cell types, including a new model cell line for oral cavity squamous cell carincoma [20], tumor initiating cells in adult human gliomas [20], [21], and IL13Rα2 expression in human pediatric brain tumors [7]. Our findings possibly explain discrepancies between publications evaluating IL13Rα2 expression. For example, when evaluating cancer stem cells, our group found that IL13Rα2 is expressed by approximately 50% of glioma stem cell lines when using the AF146 antibody [8]. However, in a separate study of glioma stem cells isolated from glioblastomas, IL13Rα2 expression was not reported when using the B-D13-PE antibody (Cell Sciences) (8 samples characterized) [22]. Our findings may also help explain inconsistencies noted in previous studies using BD-13 to evaluate IL13Rα2 expression by immunohistochemistry and flow cytometry, as compared to qPCR to measure mRNA levels [8], [23]. Our results thus highlight the necessity of re-evaluating reports using the B-D13 antibody to characterize cell surface expression of IL13Rα2.

One of the principle observations resulting from these studies is that cytokine treatment of primary low-passage glioma cell lines induces the expression of VCAM-1, expanding the previously observed cytokine induction of VCAM-1 on astrocytoma cell lines [24], as well as on other transformed and non-transformed cell types [25], [26]. As an adhesion molecule, induction of VCAM-1 on glioma cells by inflammatory cytokines is of potential clinical significance. VCAM-1 expression emerges late in tumorigenesis and is positively correlated with malignancy grades in various tumors including glioma [27]. In breast and colon cancers, VCAM-1 mediates prometastatic tumor stromal interactions, proliferation, apoptosis and invasion, all of which ultimately contribute to malignancy and tumor progression [28]. That similar processes may influence glioma progression will be the subject of future investigations.

## Supporting Information

Figure S1 **B-D13 is induced on glioma cell lines in a various cytokine conditions.** THP-1, T98, and PBT003 cells were incubated overnight with 20 ng/ml of the indicated cytokines and analyzed for cell surface expression of the B-D13 target antigen (grey histogram) vs. isotype control staining (black line).
(PDF)
