# Supplementary material for: Cytokine Induction of VCAM-1 but Not IL13Rα2 on Glioma Cells: A Tale of Two Antibodies
Source: PLoS One. 2014 May 2;9(5):e95123. doi: 10.1371/journal.pone.0095123 (PMC4008428; doi:10.1371/journal.pone.0095123)
Supplement: Figure S1 — B-D13 is induced on glioma cell lines in a various cytokine conditions. THP-1, T98, and PBT003 cells were incubated overnight with 20 ng/ml of the indicated cytokines and analyzed for cell surface expression of the B-D13 target antigen (grey histogram) vs. isotype control staining (black line). (PDF) [file pone.0095123.s001.pdf]

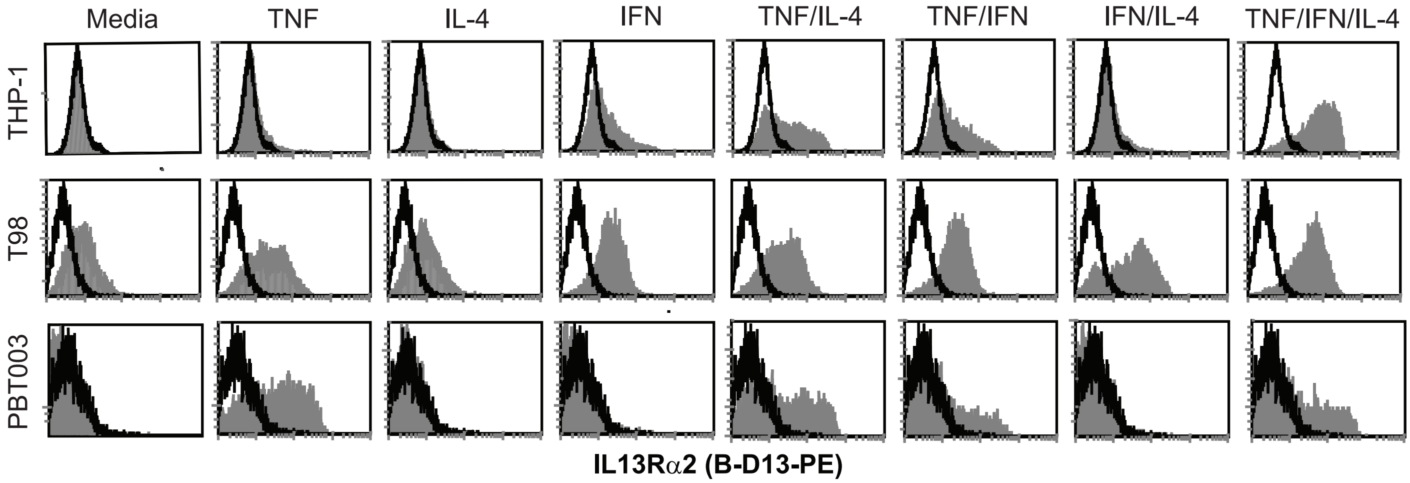

Figure S1. B-D13 is induced on glioma cell lines in a various cytokine conditions. THP-1, T98, and PBT003 cells were incubated overnight with 20ng/ml of the indicated cytokines and analyzed for cell surface expression of the B-D13 target antigen (grey histogram) vs. isotype control staining (black line).
